# Targeting the Transforming Growth Factor-β pathway inhibits human basal-like breast cancer metastasis

**DOI:** 10.1186/1476-4598-9-122

**Published:** 2010-05-26

**Authors:** Vidya Ganapathy, Rongrong Ge, Alison Grazioli, Wen Xie, Whitney Banach-Petrosky, Yibin Kang, Scott Lonning, John McPherson, Jonathan M Yingling, Swati Biswas, Gregory R Mundy, Michael Reiss

**Affiliations:** 1 Division of Medical Oncology, Department of Internal Medicine, UMDNJ-Robert Wood Johnson Medical School and The Cancer Institute of New Jersey, New Brunswick, NJ, USA; 2 Department of Molecular Biology, Princeton University, Princeton, NJ, USA; 3 Genzyme Corporation, Framingham, MA, USA; 4 Lilly Research Laboratories, Indianapolis, IN, USA; 5 Dept. of Cancer Biology, Vanderbilt Center for Bone Biology, Vanderbilt University School of Medicine, Nashville, TN, USA

## Abstract

**Background:**

Transforming Growth Factor β (TGF-β) plays an important role in tumor invasion and metastasis. We set out to investigate the possible clinical utility of TGF-β antagonists in a human metastatic basal-like breast cancer model. We examined the effects of two types of the TGF-β pathway antagonists (1D11, a mouse monoclonal pan-TGF-β neutralizing antibody and LY2109761, a chemical inhibitor of TGF-β type I and II receptor kinases) on sublines of basal cell-like MDA-MB-231 human breast carcinoma cells that preferentially metastasize to lungs (4175TR, 4173) or bones (SCP2TR, SCP25TR, 2860TR, 3847TR).

**Results:**

Both 1D11 and LY2109761 effectively blocked TGF-β-induced phosphorylation of receptor-associated Smads in all MDA-MB-231 subclones *in vitro*. Moreover, both antagonists inhibited TGF-β stimulated *in vitro *migration and invasiveness of MDA-MB-231 subclones, indicating that these processes are partly driven by TGF-β. In addition, both antagonists significantly reduced the metastatic burden to either lungs or bones *in vivo*, seemingly independently of intrinsic differences between the individual tumor cell clones. Besides inhibiting metastasis in a tumor cell autonomous manner, the TGF-β antagonists inhibited angiogenesis associated with lung metastases and osteoclast number and activity associated with lytic bone metastases. In aggregate, these studies support the notion that TGF-β plays an important role in both bone-and lung metastases of basal-like breast cancer, and that inhibiting TGF-β signaling results in a therapeutic effect independently of the tissue-tropism of the metastatic cells. Targeting the TGF-β pathway holds promise as a novel therapeutic approach for metastatic basal-like breast cancer.

**Conclusions:**

In aggregate, these studies support the notion that TGF-β plays an important role in both bone-and lung metastases of basal-like breast cancer, and that inhibiting TGF-β signaling results in a therapeutic effect independently of the tissue-tropism of the metastatic cells. Targeting the TGF-β pathway holds promise as a novel therapeutic approach for metastatic basal-like breast cancer.

## Background

In the normal mammary gland, Transforming Growth Factor-β (TGF-β) controls tissue homeostasis by inhibiting cell cycle progression, inducing differentiation and apoptosis, and maintaining genomic integrity [1-3]. In addition, TGF-β orchestrates the response to tissue injury and mediates repair by inducing epithelial-to-mesenchymal transition (EMT) and cell migration in a time-and space-limited manner [4,5]. Following extracellular activation of TGF-β, the ligand binds to the type II TGF-β receptor (TβR-II), which then recruits and activates the type I receptor (TβR-I/Alk-5)[6]. In general, the activated TβR-I/Alk-5 phosphorylates receptor-associated Smad2 and Smad3, which form complexes with Smad4. These activated Smad complexes accumulate in the nucleus where, along with co-activators and cell-specific DNA-binding factors, they regulate gene expression and ultimately cell growth and tissue repair [7,8]. More recently it has become apparent that TGF-β also activates the receptor-associated Smads1 and -5 in a TβR-I/ALK5-ALK2/3-dependent manner, and that this arm of the signaling pathway may be the predominant one driving EMT and cell migration [9-11].

Several correlative studies have suggested that the TGF-β signaling pathway plays a critical role in progression of human breast cancer. For example, there appears to be direct correlation between tumor burden and plasma TGF-β levels in patients with breast cancer [12-15]. In addition, breast cancer tissue appears to express higher levels of TGF-β than normal breast tissue [16-19]. Furthermore, a significantly greater fraction of invasive carcinomas express immunodetectable TGF-β than *in situ *carcinomas [19,20].

Besides these correlative studies, genetic manipulation of the intrinsic TGF-β signaling pathway in mammary cancer cells has provided direct evidence for its importance in driving the metastatic process (Reviewed in [21]). Thus, McEarchern et al. [22] reported that expressing a dominant negative truncated TGF-β type II receptor (*TGFBR2*) gene in highly metastatic 4T1 murine mammary carcinoma cells significantly restricted their ability to establish distant metastases. Along the same lines, Yin et al. [23] showed that expression of a dominant-negative *TGFBR2 *receptor mutant in the human MDA-MB-231breast cancer cell line inhibited the extent of experimental bone metastases. Moreover, reversal of the dominant-negative signaling blockade by overexpressing a constitutively active TβR-I receptor in these breast cancer cells increased production of parathyroid hormone-related protein (PTHrP) by the tumor cells and enhanced their osteolytic bone metastases. In similar studies, Tang et al. showed that introducing a dominant-negative *TGFBR2 *gene into highly metastatic MCF10Ca1 mammary carcinoma cells resulted in a reduction in experimental pulmonary metastases [24]. More recently, using genetic depletion experiments, several groups have demonstrated that Smad4 [25-27] as well as Smad2 and -3 [28] contribute to the formation of osteolytic bone metastases by MDA-MB-231 cells. Similarly, interference with Smad2/3 signaling strongly suppressed experimental lung metastases of aggressive MCF10Ca breast carcinoma cells [29]. In aggregate, these studies indicate that, even though human breast carcinoma cells are typically refractory to TGF-β-mediated growth suppression, the remaining intrinsic TGF-β signaling contributes to the formation of macrometastases in several different secondary sites, including bone and lungs [23-25]. These studies have generated considerable enthusiasm for exploiting the TGF-β pathway as a novel therapeutic target (reviewed in [21,30]). However, a number of key questions will need to be answered before embarking on clinical trials of TGF-β pathway antagonists in breast cancer.

First, it is necessary to validate the results of genetic depletion experiments using treatment with pharmacological inhibitors of TGF-β signaling. Currently, two main strategies for targeting TGF-β signaling are in early stages of clinical development [21,31-33]: The first involves trapping of TGF-β ligands with soluble TβR-II exoreceptor molecules [34] or with isoform-selective antibodies. These include lerdelimumab (selective for TGF-β_2_) and metelimumab (selective for TGF-β_1_), as well as the murine 1D11or humanized GC-1008 (Fresolimumab) antibodies that neutralize all three major TGF-β isoforms [33]. The second approach involves chemical inhibition of the TGF-β receptor kinases [33]. There are a number of key pharmacological and pharmacodynamic differences between these two classes of TGF-β antagonists: First, ligand traps are selective for particular ligand(s). For example, 1D11 neutralizes all 3 major active TGF-β isoforms (TGF-β1, -2, and -3)[35], but does not bind other ligands in the TGF-β superfamily, such as activins and BMPs. In contrast, most of the chemical kinase inhibitors inhibit not only Alk-5, -but also the Alk-4 and -7 kinases, thus blocking both TGF-β and activin signaling [36-39]. In addition, some of these chemicals, such as LY2109761 (Eli Lilly & Co.), target both the TβR-I and -II kinases [40]. Moreover, the neutralizing antibodies selectively inhibit biologically active TGF-βs, while the receptor kinase inhibitors also shut off the basal Smad phosphorylation that is seen in the absence of exogenously added TGF-β, so called "endogenous" signalling [41]. Finally, tissue and cell penetration of antibodies is often less efficient than of small chemicals, and the target protein needs to be accessible to the antibody to be effectively neutralized. On the other hand, chemicals have more favorable pharmacological properties than the neutralizing antibodies. Because of these differences in target specificity and pharmacological properties, it is difficult to predict which of these compounds will have superior anti-metastatic properties *in vivo*.

The second major question that needs to be addressed is whether or not metastases to different organ sites are equally dependent on TGF-β signaling. In the MDA-MB-231 model system, over-expression of a small number of genes is sufficient to selectively confer either bone-tropic or lung-tropic metastatic properties [42,43]. However, the gene expression signature associated with bone metastases is distinctly different from that associated with lung metastases, indicating that a very different type of adaptation is required for MDA-MB-231 to effectively colonize bone marrow or a pulmonary microenvironment [42]. On the other hand, several of the bone- (*IL-11*, *CTGF *and *CXCR4*) and lung metastasis genes (*GRO1/CXCL1*, *MMP-2*, *ID1*, *PTGSG2/COX2*) are regulated by TGF-β [41]. Therefore, we hypothesize that cell autonomous TGF-β signaling plays an important role in pulmonary metastases as well as in bone metastases. However, not all bone metastases may be equally dependent on autocrine TGF-β signaling. Besides rapidly growing bone metastases, some animals developed detectable skeletal metastases following a prolonged period (six months after inoculation) of dormancy (Lu et al. In Preparation) [44]. Cell lines derived from such "post-dormancy" metastases (MDA-231-2860TR and MDA-231-3847TR) retained clear bone-tropism when re-injected into animals, but they lacked expression of previously identified TGF-β-driven bone metastasis genes, such as *CXCR4 *or *IL-11 *[44]. Thus, primary lytic bone metastases may be more dependent on TGF-β signaling than the ones that develop following dormancy.

In our studies, we used 1D11, a mouse monoclonal pan-TGF-β neutralizing antibody [35] and LY2109761, a chemical inhibitor of both TβR-I and TβR-II receptor kinases [40] to determine whether or not these two antagonists have non-overlapping spectra of anti-metastatic activity against breast cancer and whether anti-metastatic activity of TGF-β pathway inhibitors varies based on tissue tropism using a human basal cell-like breast cancer model.

## Results

Several investigators have demonstrated that genetic inactivation of the TGF-β signaling pathway reduces the ability of human basal-like breast cancer cells to metastasize to bones or lungs [23-27,29]. The first question we addressed is whether treatment with pharmacological TGF-β antagonists can reproduce the effects of genetically inactivating the tumor cell autonomous TGF-β signaling pathway *in vitro *and *in vivo*. To this end, we utilized two types of TGF-β pathway antagonists, i.e. 1D11, a mouse monoclonal pan-TGF-β neutralizing antibody and LY2109761, a chemical inhibitor of TGF-β type I and II receptor kinases. We employed experimental metastasis assays in which MDA-MB-231 human breast carcinoma cells were injected either into the left cardiac ventricle to generate osteolytic bone metastases, or into the tailvein to produce pulmonary metastases. To determine whether the efficacy of the TGF-β antagonists depended on the type of metastases, we used two types of highly bone-tropic (SCP2-TR and SCP25-TR) or lung-tropic (4175-TR and 4173) subclones of MDA-MB-231 that had been isolated by *in vivo *selection [25,43,45]. In addition, during this *in vivo *selection process, some animals had developed detectable skeletal metastases only after a prolonged period (six months after inoculation) of dormancy [44]. Clonal sublines derived from such "post-dormancy" metastases, 2860TR and 3847TR, retained clear bone-tropism when re-inoculated by intracardiac injection. Because their gene expression profiles were quite distinct from the SCP lines, this allowed us to address to what extent the efficacy of TGF-β antagonists was dependent on intrinsic properties of tumor cell clones derived from the same parental line.

### Distinct morphology of MDA-MB-231 derived subclones in three-dimensional (3D) culture

**Figure 1:**
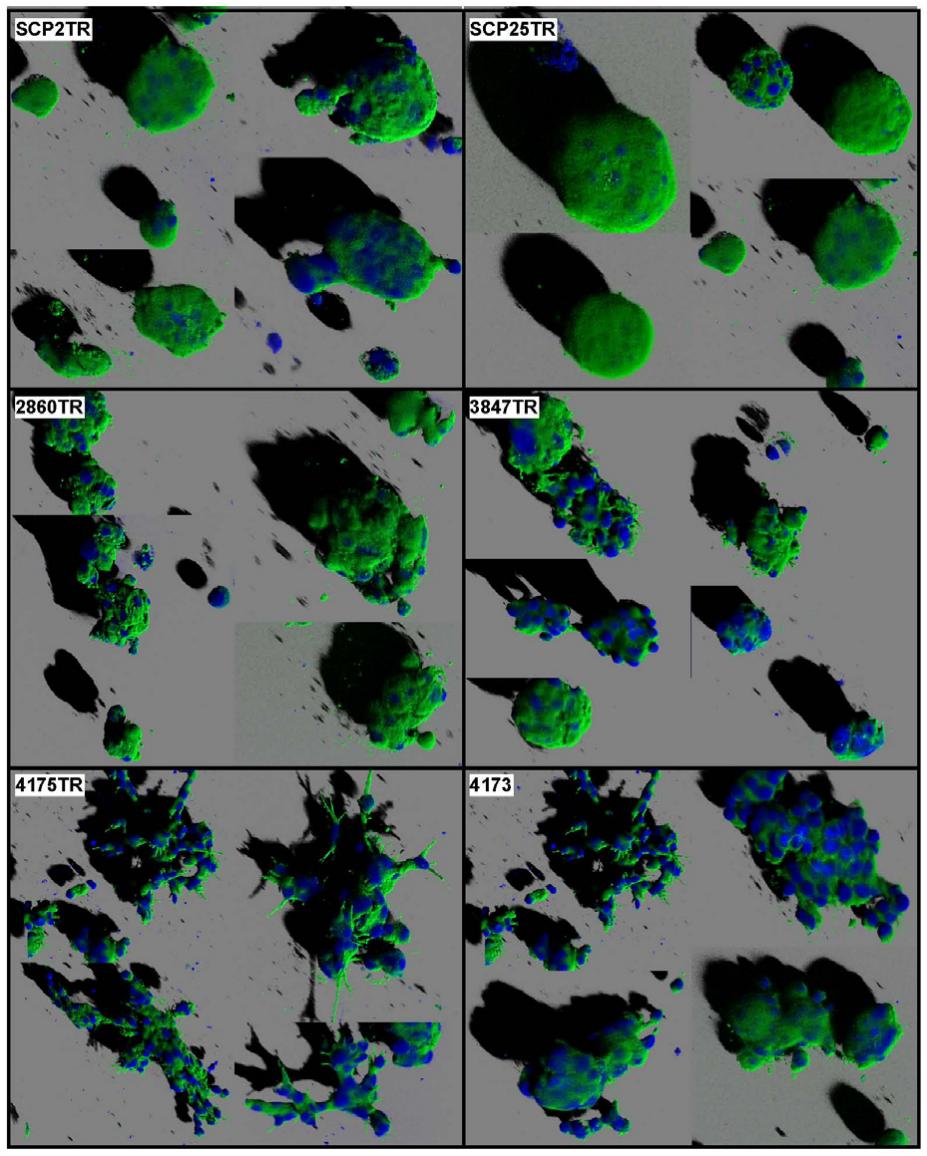
**Morphology of MDA-MB-231-derived subclones in 3D organotypic culture**. Cells were plated onto a layer of growth factor-reduced Matrigel^® ^matrix as described in Materials and Methods. Confocal microscopy images of 9 day-old 3D cultures labeled with Alexa-488-phalloidin were obtained. Colonies of early (SCP2TR and SCP25TR) and post-dormant (2860TR and 3847TR) bone-tropic subclones displayed a mass-like and grape-like morphology, respectively [46]. In contrast, the two lung-tropic subclones (4175TR and 4173) exhibited a stellate invasive phenotype [46].

Morphologically, the six MDA-MB-231 subclones were indistinguishable from each other when cultured on a plastic substratum. However, when we examined the growth patterns of the various MDA-MB-231 subclones in 3D Matrigel^® ^cultures, significant differences were noted (Figure 1). Parental MDA-MB-231 cells have previously been reported to display a stellate growth pattern in 3D culture [46]. As shown in Figure 1, the two lung-tropic MDA-MB-231 subclones, 4175-TR and 4173, largely retained this distinct stellate morphology, which was associated with pronounced invasion into the surrounding Matrigel^®^. In contrast, the two bone-tropic subclones, SCP2TR and SCP25TR, displayed a mass-like phenotype, while colonies formed by the two post-dormant subclones, 2860TR and 3847TR, displayed a looser, so-called grape-like, phenotype (Figure 1)[46]. Thus, each of the three clonal subsets displayed a distinct growth pattern in this 3D culture environment, presumably reflecting intrinsic differences in gene expression profiles and their unique metastatic properties *in vivo *[25,43-45].

### Effects of TGF-β antagonists on Smad activation in MDA-MB-231 cell clones *in vitro*

**Figure 2:**
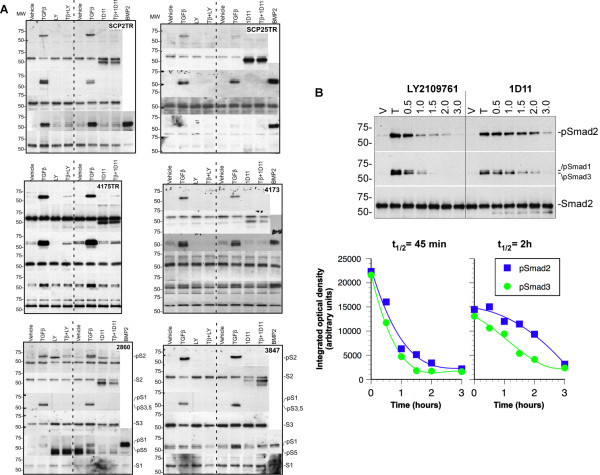
**Effects of TGF-β antagonists on Smad activation in MDA-MB-231-derived metastatic subclones. A**. Cells were starved in serum free DMEM medium overnight and incubated with vehicle or TGF-β antagonist for 15 minutes. Subsequently, TGF-β (100 pM) was added, and cells were incubated for an additional hour. The levels of phosphorylated Smad-2,-3 and -1/5/8 were determined by Western blotting. Induction of Smad-2 and -3 phosphorylation by exogenous TGF-β was effectively inhibited by either LY2109761 (2 μM) or 1D11 (10 μg/ml) in all six subclones. TGF-β induced Smad1 and -5 phosphorylation most strongly in the most metastatic bone-tropic (SCP2TR) and lung-tropic (4175TR and 4173) subclones, and to a lesser extent in the 2860TR and 3847TR cells. Activation of these BMP Smads was also inhibited by both antagonists. **B**. To assess the ability of TGF-β antagonists to induce dephosphorylation of R-Smads, bone-tropic SCP2TR cells were treated with TGF-β for 1.5 h, followed by treatment with the antagonists for the indicated time-periods. The receptor kinase inhibitor, LY2109761, induced dephosphorylation of activated Smad-2 and -3 more rapidly than the pan- TGF-β neutralizing antibody, 1D11.

Since activation of receptor associated Smads (R-Smads) is a required step in TGF-β signaling, we examined the effects of treatment with TGF-β antagonists on TGF-β-induced Smad phosphorylation. As shown in Figure 2A, TGF-β treatment induced phosphorylation of Smad2 and -3 in each of the six cell lines. In addition, TGF-β clearly induced phosphorylation of Smad-1 and -5 in the highly metastatic SCP2TR, 4175TR and 4173 clones, to a much lesser extent in the two post dormancy clones (2860TR and 3847TR), and not at all in the moderately metastatic SCP25TR cells (Figure 2A). These findings suggest that the degree of Smad1 and -5 activation may reflect the intrinsic metastatic ability and/or tissue tropism of the different MDA-MB-231 subclones.

Pretreatment of cells with either the TβR-I and TβR-II dual kinase inhibitor, LY2109761, or the pan-TGF-β neutralizing murine antibody, 1D11, effectively inhibited TGF-β-induced activation of all R-Smads. Given the different pharmacological properties of the two compounds, we also examined their effects on Smad signal termination. Treatment of SCP2TR cells with LY2109761 induced dephosphorylation of Smad2 and -3 much more rapidly (t_1/2_= 45 minutes) than 1D11 (t_1/2 _= 2 h)(Figure 2B). Thus, while both LY2109761 and 1D11 were equally capable of blocking TGF-β-induced signal activation, the kinetics with which they terminated TGF-β signaling were quite distinct.

### Effects of TGF-β antagonists on cell proliferation migration and invasion of MDA-MB-231 clones *in vitro*

**Figure 3:**
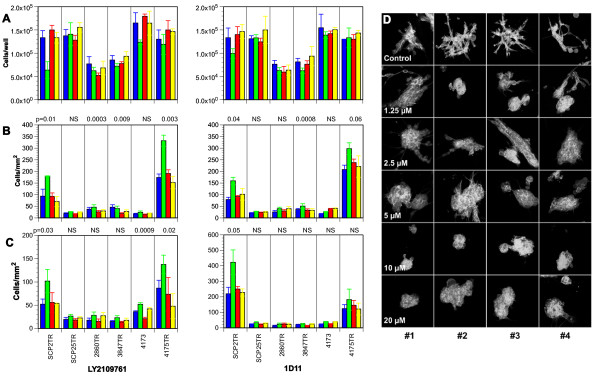
**Effects of TGF-β and TGF-β antagonists on cell growth and cell-motility and -invasion of MDA-MB-231 subclones**. **A**. Cells were plated at 2 × 10^4^/well in 24-well plates and incubated in the presence of vehicle (Blue bars), TGF-β (100 pM) (Green bars), 1D11 (10 μg/ml) or LY2109761 (2 μM) (Red bars) or a combination of TGF-β and an inhibitor (Yellow bars) for 72 h and cell numbers determined. Treatment with exogenous TGF-β failed to significantly inhibit growth of any of the subclones, with the exception of SCP2TR cells. Neither 1D11 nor LY2109761 stimulated tumor cell growth of any of the MDA-MB-231 subclones. Means ± SD of at least three independent experiments. Unpaired 2-sided t-test ± Welch correction was used to compare treatment with or without TGF-β antagonist. For cell-motility (**B) **and -invasion **(C) **assays, MDA-MB-231 sublines were cultured in uncoated and Matrigel^®^-coated PET inserts, respectively. Cells were treated with TGF-β (100 pM) and either LY2109761 (2 μM) or 1D11 (10 μg/ml) for 24 h. MDA-MB-231 subclones SCP2TR and 4175TR displayed the greatest motility and invasion, which were further stimulated by exogenous TGF-β. Moreover, both TGF-β pathway antagonists significantly inhibited TGF-β-induced motility and invasion in these two cell lines. Means ± SD of at least three independent experiments. Unpaired 2-sided t-test ± Welch correction was used to compare treatment with TGF-β alone with TGF-β plus inhibitor. **D**. Lung-tropic 4173 cells were plated onto a layer of growth factor-reduced Matrigel^® ^matrix, followed by treatment with either vehicle or varying concentrations of LY2109761 for 9 days. Phase contrast microscopy images of 9 day-old 3D cultures labeled with Alexa-488-phalloidin were obtained. Numbers refer to the four representative colonies from each culture shown. As can be seen, treatment with LY2109761 inhibited invasion into surrounding Matrigel^® ^in a dose-dependent manner, resulting in a reversal from a stellate to mass-like phenotype.

Treatment with exogenous TGF-β failed to significantly affect the growth of MDA-231-4175TR, -4173, -SCP25TR, -2860TR and -3847TR cells *in vitro *(Figure 3A). Moreover, even though TGF-β inhibited SCP2TR cell growth by 30% and this reached statistical significance (p = 0.029), this was far less than in non-neoplastic cells [39]. Most importantly, neither of the two TGF-β pathway antagonists significantly stimulated growth of any of the six MDA-MB-231 clones (Figure 3A). Previous studies have suggested that basal cell-like breast cancer invasion and migration might be driven by TGF-β [47]. Hence, we determined the effects of each of the antagonists on tumor cell motility and invasion *in vitro*. As shown in Figures 3B and 3C, the MDA-MB-231 subclones differed markedly in terms of intrinsic motility and invasiveness, with SCP2TR and 4175TR being the most motile and invasive. Moreover, exogenous TGF-β most strongly stimulated *in vitro *migration and invasion of these two MDA-MB-231 clones. Interestingly, neither antagonist seemed to have a significant effect on the basal migration rates of any of the subclones (Figure 3B, C). However, treatment with either LY2109761 or 1D11 effectively counteracted TGF-β-induced migration as well as invasion of SCP2TR and 4175TR cells *in vitro*. Finally, neither antagonist affected the intrinsic invasion rates of these cell lines in Transwell^® ^assays, with the exception of 4173 cells (Figure 3C). Consistent with these findings, treatment of lung-tropic MDA-MB-231 4173 cells in 3-dimensional Matrigel^® ^cultures with LY2109761 inhibited spontaneous invasion and caused the cells to revert to a mass-like growth pattern in a dose-dependent manner (Figure 3D). These findings suggested that the invasive properties of MDA-MB-231 4173 colonies in 3D cultures are dependent on autocrine TGF-β signaling.

Because SCP2TR and 4175TR cells displayed the highest basal migration and invasion rates, were most strongly stimulated by TGF-β, and were most susceptible to both TGF-β pathway antagonists, these two MDA-MB-231 subclones were selected for *in vivo *studies.

### Effects of TGF-β antagonists on bone metastases *in vivo*

**Figure 4:**
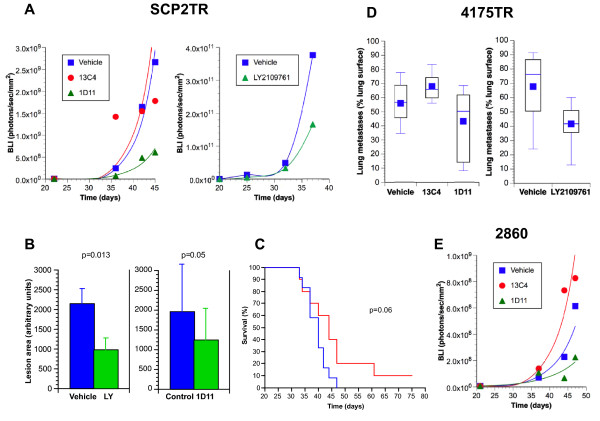
**Effects of TGF-β antagonists on experimental MDA-MB-231 human breast cancer cell metastases *in vivo***. **A**. Mice were inoculated with bone-tropic SCP2TR cells via intracardiac injection and treated with vehicle, 13C4 isotype control antibody or 1D11 anti-TGF-β antibody (**Left panel**) or with vehicle or LY2109761 (**Right panel**). Bone metastases were monitored by BLI once weekly. Median values per group are shown. Treatment with 1D11 antibody reduced the burden of bone metastases by approximately 70-80% (p = 0.001) compared to treatment with either vehicle or isotype control antibody. Similarly, LY2109761 treatment inhibited bone metastases compared to vehicle controls by approximately 55% (p = 0.039). **B**. Faxitron analysis of fore-and hind limbs of tumor-bearing animals. Both 1D11 and LY2109761 treatment resulted in significant reductions in the total extent of SCP2TR-induced osteolytic bone lesions. Unpaired 2-sided t-test was used for comparisons. **C**. Treatment of bone-tropic SCP2TR inoculated mice with the 1D11 antibody was associated with prolongation of survival of the test animals (n = 10) compared to control animals (n = 12) (p = 0.06, Log-Rank test). **D**. Mice were inoculated with lung-tropic 4175TR cells via tailvein injection and treated with vehicle, 13C4 or 1D11 (**Left panel**) or with vehicle or LY2109761 (**Right panel**). Treatment with 1D11 antibody reduced the metastatic burden to lungs by approximately 25-40% (p = 0.001, Kruskall-Wallis test) compared to treatment with either vehicle or isotype control antibody. Similarly, LY2109761 treatment reduced the burden of lung metastases compared to vehicle by approximately 40% (p = 0.079). **E**. Treatment of mice inoculated with post dormancy bone tropic 2860TR cells with 1D11 antibody reduced the metastatic burden to bones by between 55-80% compared to treatment with vehicle or isotype control antibody (p = 0.019).

Several studies have demonstrated that tumor cell autonomous genetic inactivation of the TGF-β signaling pathway by knock-down of *TGFBR2 *or *SMAD4 *reduced the ability of MDA-MB-231 human basal-like breast cancer cells to metastasize to bone [23,25-27]. Whether these effects could be reproduced by treatment with TGF-β antagonists was determined in experimental metastasis assays in which we inoculated athymic nude mice with bone-tropic SCP2TR cells via intracardiac injection. In separate experiments, mice were treated with 5 mg/kg 1D11 given intraperitoneally (i.p.) three times per week or with 50 mg/kg LY2109761 twice daily by gavage, beginning 1-3 days following tumor cell inoculation. No drug-associated toxicities were observed and animals maintained their body weight during the entire course of treatment (data not shown). Because the tumor cell lines expressed a luciferase reporter construct, metastases could be monitored *in vivo *using bioluminescence imaging (BLI) (Figure 4A). Treatment with 1D11 antibody reduced the burden of bone metastases by approximately 70-80% (p = 0.001) compared to treatment with either vehicle or isotype control antibody (Figure 4A). Similarly, LY2109761 treatment inhibited bone metastases compared to vehicle controls by approximately 55% (p = 0.039) (Figure 4A). Results obtained by BLI were confirmed post mortem using Faxitron analysis (Figure 4B). Perhaps most importantly, treatment with the 1D11 antibody as a single agent was associated with a trend towards prolongation of survival of the test animals (Figure 4C).

### Effects of TGF-β antagonists on pulmonary metastases *in vivo*

To address the question whether TGF-β signaling plays a similar role in pulmonary metastases as in bone metastases, mice were inoculated with lung-tropic 4175TR cells via tail vein injection (Figure 4D). In separate experiments, mice were then treated either with 5 mg/kg 1D11 given intraperitoneally (i.p.) three times per week or with 50 mg/kg LY2109761 twice daily by gavage, beginning 1-3 days following tumor cell inoculation. Treatment with 1D11 antibody reduced the metastatic burden to lungs by approximately 25-40% (p = 0.001) compared to treatment with either vehicle or isotype control antibody (Figure 4D). Similarly, LY2109761 treatment reduced the burden of lung metastases compared to vehicle by approximately 40% (p = 0.079) (Figure 4C). These results indicate that the establishment of pulmonary metastases is also, at least in part, dependent on TGF-β signaling. As was the case with bone metastases, the fact that both neutralization of TGF-β itself and selective chemical inhibition of the type I and -II TGF-β receptor kinases had similar effects in inhibiting pulmonary metastases is indicative of a specific role for TGF-βs (as opposed to activins or BMPs) in this process.

### Effect of 1D11 on primary versus post-dormant bone metastases *in vivo*

MDA-MB-231 bone tropic subclones derived from "post-dormancy" bone metastases (2860 TR and 3847 TR) have a distinct gene expression that does not include the previously identified bone metastasis gene signature (Lu et al. In Preparation)[44]. These differences between "primary" and "post dormant" bone-tropic MDA-MB-231 clones allowed us to address to what extent the efficacy of TGF-β antagonists might differ as a function of intrinsic properties of tumor cell clones derived from the same parental line. Mice were inoculated with post dormant bone tropic 2860 TR cells via intracardiac injection. Treatment with 1D11 antibody reduced the metastatic burden to bones by between 55-80% (p = 0.019) compared to treatment with vehicle or isotype control antibody (Figure 4E). Thus, TGF-β neutralizing antibody 1D11 inhibited bone metastases from 2860 TR cells to a similar degree as those from SCP2TR cells (Figure 4A). In aggregate, the anti-metastatic activity of TGF-β targeted agents appears to be relatively independent of the intrinsic differences in gene expression signatures of individual subclones.

### Molecular target inhibition by TGF-β antagonists *in vivo*

**Figure 5:**
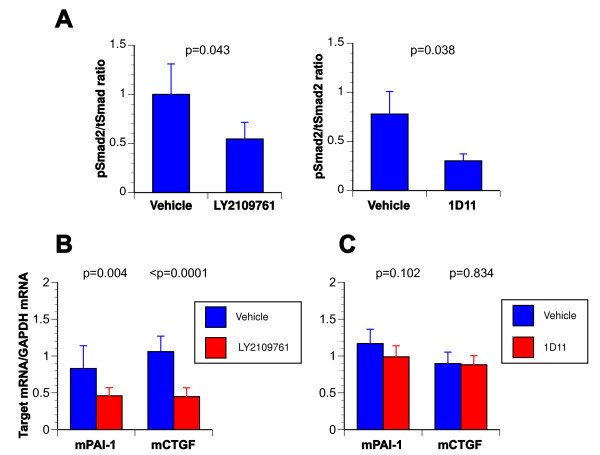
**Pharmacodynamic effects of TGF-β antagonists *in vivo***. **A**. Protein extracts from snap-frozen uninvolved lung tissue of mice treated with vehicle and LY2109761 and of mice treated with controls and 1D11 were prepared and subject to Western blotting using rabbit phosphoSmad2 antibody (1:1000 dilution). Whole cell lysate from SCP2TR was used as a control (Co). Treatment with both antagonists resulted in a reduction of phosphorylated Smad2 levels. Values represent the means and SD of three mice per group. Unpaired 2-sided t-test was used for comparisons. **B**. RNA was extracted from snap-frozen uninvolved kidneys of mice treated with vehicle or LY2109761 using Trizol reagent (Invitrogen) and purified using RNeasy mini columns (Qiagen) according to the manufacturer's instructions. Transcript levels of CTGF and PAI-1 were assayed using the QuantiTect™ Probe RT-PCR Kit on a Mx4000^® ^Multiplex Quantitative PCR System (Stratagene). Treatment with LY2109761 significantly reduced mRNA levels of both CTGF and PAI-1 relative to GAPDH mRNA. Values represent the means and SD of three mice per group. Unpaired 2-sided t-test was used for comparisons. **C**. RNA was extracted from snap-frozen lungs of mice treated with vehicle, isotype control antibody or 1D11 and transcript levels determined as described above. No significant reduction in CTGF or PAI-1 mRNA levels could be detected in the 1D11-treated group. Values represent the means and SD of two independent experiments, 3 mice per group. Unpaired 2-sided t-test was used for comparisons.

To substantiate the inhibition of TGF-β signaling by 1D11 or LY2109761 treatment *in vivo*, we ascertained the levels of phospho-Smad2 in uninvilved lung tissue and mRNA of several TGF-β target genes in kidney tissue of treated animals. Phospho-Smad2 levels were reduced compared to vehicle controls in protein extracts from lungs of animals treated with either LY2106791 or 1D11 (Figure 5A). As shown in Figure 5B, LY2109761 treatment significantly lowered basal CTGF and PAI-1mRNA expression levels, consistent with blockade of endogenous TGF-β signaling *in vivo*. In contrast, basal TGF-β target genes transcript levels were not affected by 1D11 treatment (Figure 5C), suggesting that this agent may selectively spare endogenous TGF-β signaling [41].

### Mechanisms of action of TGF-β antagonists *in vivo*

**Figure 6:**
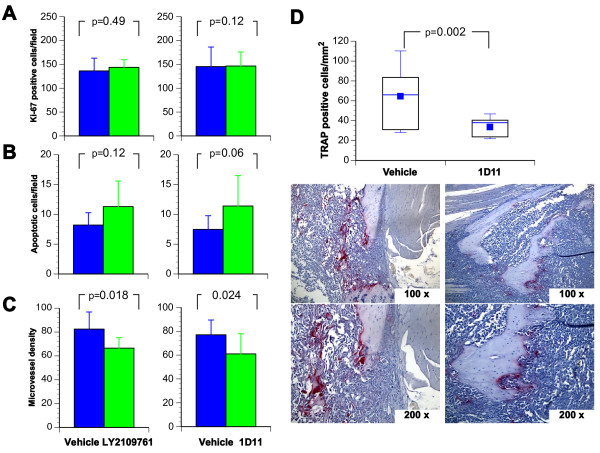
**Mechanism of action of TGF-β antagonists *in vivo***. Lung metastases from mice treated with LY2109761 and 1D11 were stained for markers of cell proliferation (Ki-67), apoptosis (TUNEL), and microvascular endothelial cells (CD34). At least 600 nuclei were counted in 5 randomly selected high-power (400 ×) fields in areas of viable tumor to determine the proportion of Ki-67-or TUNEL-positive cells. The total number of CD34^+ ^microvessels were counted in 5 randomly selected high-power (400 ×) fields in areas of viable tumor. **A**. Neither antagonist affected tumor cell proliferation (Ki-67; p = 0.49 for the LY2109761 group and p = 0.12 for the 1D11 group) or **B**. apoptosis (TUNEL assay; p = 0.12 for the LY2109761 group and p = 0.06 for the 1D11 group). **C**. However, microvessel density was significantly reduced in tumors of mice treated with LY2109761 and 1D11 compared with their respective controls (p = 0.018 for the LY2109761 group and p = 0.024 for the 1D11 group). **D**. Histological staining for tartrate resistant acid phosphatase (TRAP) activity (red color) of bone metastases from representative vehicle-(left images) and 1D11-treated (right images) mice. The numbers of TRAP positive cells per mm^2 ^of tumor adjacent to bone in bone metastases from 1D11-treated mice (n = 9 lesions) relative to vehicle-treated mice (n = 17 lesions) are shown. Treatment with 1D11 was associated with a significant reduction in the number of TRAP-positive osteoclasts (p = 0020). Unpaired 2-sided t-test (± Welch correction) was used for comparisons.

In order to assess possible mechanisms of action of the two TGF-β antagonists on metastases *in vivo*, we compared the rates of tumor cell proliferation and apoptosis between metastases in the different treatment groups. Consistent with our *in vitro *results, neither antagonist had a significant effect on tumor cell proliferation (Ki67 staining, Figure 6A) or apoptosis (TUNEL staining, Figure 6B). In contrast, treatment with either 1D11 or LY2109761 resulted in a significant reduction in microvessel density in lung metastases as determined by CD34 staining (Figure 6C). This suggested that these compounds act, at least in part, by inhibiting tumor angiogenesis. These findings were entirely consistent with our previous findings using a murine model of metastatic mammary cancer treated with a different selective TGF-β type I receptor kinase inhibitor [48]. As shown in Figure 4, both 1D11 and LY2109761 treatment resulted in significant reductions in osteolytic bone lesions. Consistent with this, histological staining for tartrate resistant acid phosphatase (TRAP) activity, a marker of active osteoclasts, showed that treatment with 1D11 significantly reduced the number of TRAP-positive osteoclasts located at the tumor:bone interface (Figure 6D). In summary, in our xenograft mouse models, the anti-metastatic properties of TGF-β signaling antagonists appear to be mediated both by tumor cell autonomous effects and by modulating tumor:host interactions via several different mechanisms, including inhibition of angiogenesis in the case of lung metastases and inhibition of osteoclast activity in the case of bone metastases.

## Discussion

Our study clearly demonstrates that treatment with TGF-β antagonists inhibits the ability of bone-as well as lung-tropic MDA-MB-231 cell lines to establish experimental metastases *in vivo*. This convincingly demonstrates that TGF-β signaling plays an important role in this process, largely independently of the organo-tropism of the tumor cells (Figure 4). Our results are consistent with several previous studies that have reported anti-metastatic activity of individual TGF-β antagonists in *in vivo *models of human mammary cancer. For example, Arteaga et al. [49] reported that intraperitoneal injections of the murine TGF-β neutralizing antibody, 2G7 (Genentech^®^), was able to suppress lung metastases of MDA-MB-231 breast cancer cells that had been inoculated intraperitoneally. More recently, using the same experimental metastasis assay we employed, Ehata et al. [50] reported that treatment with a TGF-β type I receptor kinase inhibitor, Ki26894, decreased bone metastases and prolonged survival of mice inoculated with highly bone-tropic human MDA-MB-231-D breast cancer cells. Similarly, Korpal et al. [27] recently reported that treatment with LY2106791 inhibited early skeletal metastases.

In our hands, both classes of TGF-β antagonist significantly reduced the burden of skeletal and pulmonary metastases (Figure 4). Prior to our study, little information was available to determine whether the anti-metastatic efficacy of TGF-β antagonists on human breast carcinoma was organ site-specific. Separate reports indicated that the anti-TGF-β antibody 1D11 appeared to inhibit skeletal-or pulmonary metastases of the murine 4T1 mammary carcinoma cells. Thus, treatment with 1D11 resulted in a significant reduction in the number of 4T1 lytic bone lesions [51]. Using the same 4T1 cell line, Nam et al. showed that treatment with 1D11 significantly suppressed both the number and size of tumor metastases to the lungs [52-54]. Although one has to be cautious about direct comparisons across studies, the therapeutic effects of TGF-β neutralizing antibodies against 4T1-derived skeletal or pulmonary metastases appeared to be of a similar order of magnitude.

Although our results are consistent with previous reports of anti-metastatic activity of individual TGF-β antagonists in *in vivo *breast cancer models, none of the previous studies have conducted a comparison between two different pharmacological strategies to inhibit TGF-β signaling. Thus, our second most important finding is that both neutralization of active TGF-βs using the 1D11 antibody and inhibition of TGF-β receptor kinases using the dual receptor kinase inhibitor, LY2109761, resulted in quantitatively remarkably similar degrees of inhibition of experimental metastases to both bone and lungs. Besides inhibiting the TGF-β type I (and -II) receptor kinases, LY2109761 also inhibits the activin receptor kinases, Alk-4 and Alk-7. This is a property shared by all known other members of this class of compounds, raising the concern that their biological activity may be mediated by either TGF-βs or activins. On the other hand, 1D11 is specific for bioactive TGF-βs and does not neutralize any of the other TGF-β superfamily members, including activin or BMPs. Thus, the qualitatively and quantitatively similar anti-metastatic effects we observed using both compounds in both experimental metastasis assays strongly support a specific role for TGF-β in this process, and essentially exclude the possibility that the effects we observed were due to interference with either activin-or BMP signaling.

*In vitro*, treatment with exogenous TGF-β induced Smad2/3 phosphorylation in all six MDA-MB-231 subclones and both TGF-β antagonists were capable of blocking Smad2/3 signal activation (Figure 2). In addition, both compounds effectively cause Smad2/3 signal termination, albeit that LY2109761 induced dephosphorylation of Smad2 and -3 more rapidly than 1D11. Consistent with these *in vitro *findings, *in vivo*, phospho-Smad2 levels were reduced in lungs of animals treated with either compound compared to vehicle treated controls (Figure 5). Moreover, LY2109761 treatment partly inhibited mRNA expression of TGF-β target genes, consistent with blockade of endogenous TGF-β signaling *in vivo*. These results are consistent with our previous findings using the TGF-β type I receptor inhibitor, SD-208, in the syngeneic 4T1 mammary cancer model [48]. In contrast, 1D11 treatment was not associated with a significant reduction in target gene transcript levels by *in vivo*, suggesting that this agent only neutralizes activated ligand and selectively spares endogenous TGF-β signaling.

We and others have recently reported that, besides Smad2 and -3, TGF-β also activates the BMP Smads, Smad1 and -5, in normal and malignant mammary and epidermal epithelial cells [9-11,55,56]. Moreover, the degree to which exogenous TGF-β induced Smad1/5 phosphorylation in the different subclones appears to reflect their metastatic ability *in vivo *(Figure 2). Thus, the activation state of BMP Smads should be explored as a predictive biomarker of response to TGF-β antagonists in a clinical setting.

A major unresolved question is whether and under which conditions the predominant role TGF-β plays is mediated by its tumor cell autonomous effects, or via its actions on the host microenvironment. We approached this question by comparing two types of bone-tropic MDA-MB-231 subclones. Following intracardiac inoculation with MDA-MB-231 cells, some animals developed skeletal metastases following a prolonged period of dormancy (Lu et al., In Preparation). Cell lines derived from these "post-dormancy" metastases retain clear bone-tropism when re-injected into secondary animals, but display a gene expression profile that is quite distinct from that found in the "primary" bone metastases (Lu et al. In Preparation) [44]. However, when we treated mice that had been inoculated with post-dormancy bone tropic 2860 TR cells with the 1D11 TGF-β neutralizing antibody, the development of skeletal metastases was inhibited to a similar extent as in SCP2-TR inoculated mice (Figure 4). Thus, 1D11 appeared to be anti-metastatic independently of the intrinsic gene expression profile of individual bone tropic tumor cell clones derived from the same parental cell line. These results suggest that, at least in this MDA-MB-231 *in vivo *model, TGF-β's pro-metastatic activity may be mediated predominantly by its actions on host cells within the bone microenvironment, rather than by autocrine effects on the tumor cells themselves. Consistent with this idea, neither LY2109761 or 1D11 treatment inhibited tumor cell proliferation or induced tumor cell apoptosis, *in vivo *(Figure 6).

In response to activated TGF-β released from bone matrix, MDA-MB-231 cells secrete a number of signaling molecules, including PTHrP and RANK-L, that stimulate osteoclast activity [23]. Osteoclast-mediated bone breakdown is thought to release TGF-β, thereby resulting in a "vicious cycle" that leads to progressive bone destruction [57]. Thus, we predicted that treatment with TGF-β antagonists would decrease osteoclast activation in the context of MDA-MB-231 bone metastases. In fact, 1D11 treatment resulted in a significant reduction in the number of active osteoclasts at the tumor:bone interface (Figure 6). Similarly, Futakuchi et al. [57] recently reported that treatment with 1D11 inhibited osteoclast activation and osteolytic bone destruction by 4T1 mammary carcinoma cells *in vivo*. In this study, identical effects were obtained using a chemical TGF-β type I receptor kinase inhibitor [57]. Consistent with these findings, Mohammad et al. [58] recently reported that treatment with the TGF-β type I receptor kinase inhibitor, SD-208, increased osteoblast differentiation and bone formation, while reducing osteoclast differentiation and bone resorption. In aggregate, these studies have clearly demonstrated that pharmacological blockade of TGF-β signaling shifts the balance from bone breakdown to bone (re)generation, thereby inhibiting tumor-associated osteolysis.

In the lung metastasis model, treatment with TGF-β pathway antagonists inhibited tumor angiogenesis, as reflected by a decrease in CD34-positive microvessel density. These findings are consistent with our own earlier studies of the effects of the TβR-I kinase inhibitor, SD-208, against 4T1 lung metastases [48]. Similarly, Nam et al. [54] reported that treatment with 1D11 was associated with a statistically significant decrease in microvessel density in 4T1 murine mammary tumors. Consistent with these findings, treatment of 4T1 tumor bearing mice with the 2G7 anti-TGF-β neutralizing antibody significantly reduced circulating VEGF levels [59](Genentech, US Patent Application 2005/0276802 A1). Thus, at least in lung metastases, TGF-β pathway antagonists have been consistently found to exert modest anti-angiogenic effects against basal-like mammary cancer *in vivo*.

Even though both TGF-β antagonists clearly had a demonstrable anti-metastatic effect in the MDA-MB-231 human breast cancer models, neither of the two agents completely abolished skeletal or pulmonary metastases. In part, this may be due to the fact that we had to use immunodeficient mice as hosts for human tumor cells because TGF-β pathway antagonists have been shown to de-repress anti-tumor immunity in mouse models of mammary cancer [48,49,52-54]. For example, we ourselves demonstrated that treatment with the TGF-β type I receptor kinase inhibitor, SD-208, inhibited spontaneous pulmonary metastases of R3T mammary carcinoma cells much more strongly in syngeneic than in nude mice [48]. Published studies have demonstrated that tumor-associated TGF-β not only suppresses NK cell activity and T-cell mediated anti-tumor responses, but also actively subverts the CD8^+ ^arm of the immune system into directly promoting tumor growth by an IL-17-dependent mechanism [48,49,52-54]. As we utilized athymic nude mice as hosts, we cannot ascribe the observed anti-metastatic effects of TGF-β antagonists to stimulation of T-cell-dependent processes. Moreover, even though Arteaga et al. were able to detect an effect on NK cells, even in the MDA-MB-231 model [49], we were unable to detect an increase in NK cell infiltration into metastases of 1D11 or LY2109761 treated animals in the current study (data not shown). Thus, we predict that treatment with TGF-β antagonists will have significantly greater anti-metastatic impact when applied in the context of a syngeneic host, in which they will act by a cooperative mechanism that involves several different cellular compartments, including the CD8^+ ^T cells, NK cells, the microvasculature, osteoclasts and the tumor cells themselves [54].

Finally, we should note that all of the pre-clinical studies of TGF-β pathway antagonists in mammary cancer reported to date, have employed cell lines derived from basal-like tumors. Thus, these studies preclude any conclusions regarding the possible anti-metastatic activity these compounds may or may not have in the context of estrogen-dependent or HER2-mediated breast cancers. In fact, a wealth of experimental and clinical evidence suggests that, as long as breast cancers remain dependent on estrogens, TGF-β protects against rather than promotes tumor progression [21]. Thus, one has to be cautious in extrapolating the results from the current and other preclinical studies of TGF-β pathway antagonists to breast cancers other than those of the basal-like subtype.

## Conclusions

In summary, pre-clinical studies in several different syngeneic as well as allogeneic mammary cancer models have provided convincing evidence that targeting the TGF-β pathway using either a TGF-β neutralizing antibody or receptor kinase inhibitors can inhibit both early lung and bone metastases of basal-like breast cancer. Our findings are consistent with the concept that TGF-β signaling plays several different roles in the complex interplay between tumor and host cells that constitute the pre-metastatic niche. The signaling pathway appears to be fundamentally altered in tumor cells in such a way that the tumor cells interpret incoming signals as pro-invasive, while they are no longer growth inhibited. This results in the secretion of TGF-β-induced metastasis-effector proteins, which exert pro-metastatic actions on the host microenvironment. Our studies provide substantive support for clinical trials of TGF-β antagonists for patients with basal-like breast cancer.

## Methods

### Reagents

Human recombinant TGF-β1 (1 μg/mL; Austral Biologicals, San Ramon, CA) was dissolved in 4 mmol/L HCl and 1 mg/mL bovine serum albumin (Sigma, St. Louis, MO). 1D-11 and the isotype-matched murine IgG1 monoclonal control antibody, 13C4, directed against Shigella toxin, (Genzyme, Framingham, MA) was diluted in formulation buffer composed of 0.1 M glycine, 70 mM Na_2_HPO_4_, 0.0011% Tween 20 for both *in vitro *and *in vivo *studies. A 10 mM stock solution of LY2109761 (Eli Lilly and Co., Indianapolis, IN) in DMSO (Sigma, St. Louis, MO) was prepared for *in vitro *studies. For *in vivo *studies, LY2109761 was suspended in a formulation composed of 1% sodium carboxy methylcellulose (NaCMC), 0.5% sodium lauryl sulfate (SLS), 0.05% antifoam and 0.085% polyvinylpyrrolidone (PVP).

### Cell culture

MDA-231-SCP2TR, MDA-231-SCP25TR, MDA-231-2860TR and MDA-231-3847TR are clonal sublines of MDA-MB-231 (ATCC) human breast carcinoma cells with distinct organ-specific metastatic behavior that were generated by one of us (YK)[25,42]. MDA-231-4175TR and MDA-231-4173 were obtained from Dr. Joan Massagué (Sloan Kettering Institute, New York, NY). All MDA-MB-231 sublines were maintained in DMEM (Invitrogen, Carlsbad, CA) supplemented with 10% FBS (Sigma, St Louis, MO).

### Cell proliferation assays

Cells were plated at 2 × 10^4 ^cells/well in 24 well cluster dishes (Corning Inc. Corning, NY), overnight. Cells were treated initially with 10 μg/ml 1D11 or 2 μM LY2109761 for 15 minutes followed by addition of 100 pM TGF-β1 and incubated at 37°C for 72 h. Subsequently, cells were washed with 1 ml ice-cold PBS, and detached with 0.2 ml trypsin-EDTA (Invitrogen, Carlsbad, CA). Trypsin was neutralized by adding 0.8 ml of the culture medium containing 10% FBS, and the cells counted using a Vi-cell particle Counter (Beckman Inc, Miami, FL).

### Western blot analysis

To determine the effects of TGF-β antagonists on TGF-β-induced R-Smad phosphorylation, MDA-MB-231 sublines were incubated in serum free medium overnight and treated with 2 μM LY2109761 or 10 μg/ml 1D11for 15 minutes, followed by the addition of 100 pM TGF-β1 for one hour. The vehicle control, DMSO, was used at a final concentration of 0.01%, which was not toxic to cells. For dephosphorylation assays, cells were initially treated with 100 pM TGF-β for 1.5 hour followed by three washes with serum free medium. Subsequently, cells were treated with either 2 μM LY2109761 or 10 μg/ml 1D11 for 0.5, 1, 1.5, 2 and 3 hours. Cells were then lysed *in situ *using buffer composed of 150 mM NaCl, 10 mM Tris-HCl (pH 8.0), 1 mM EGTA, 1% (v/v) Triton-X-100 in the presence of protease inhibitors and phosphatase inhibitors (Complete Mini Protease Inhibitor Cocktail Tablets with EDTA, and PhosSTOP, Roche Diagnostics Corporation, Indianapolis, IN), for 30 min at 4°C. Cell lysates were collected and clarified by centrifugation at 12,000 rpm for 10 minutes at 4°C. The clarified lysates were then subjected to SDS-PAGE and transferred to nitrocellulose membranes using a Panther™ Semidry Electroblotter (Owl Separation Systems, Portsmouth, NH). Activated Smad2 (pSmad2), Smad3 (pSmad3) and Smad1/5/8 (pSmad1/5/8), were detected using monoclonal rabbit anti-human pSmad2, polyclonal rabbit anti-human pSmad3 and polyclonal rabbit anti-human pSmad1/5/8 antibodies (Cell Signaling, Danvers, MA) at 1:1000 dilutions. Total Smad2, Smad3 and Smad1 were detected using mouse monoclonal anti-human Smad2 (Cell Signaling, Danvers, MA), rabbit monoclonal anti-human Smad3 (Zymed Laboratories, South San Francisco, CA) and rabbit monoclonal anti-human Smad1 (Cell Signaling, Danvers, MA) antibodies at 1:400, 1:500, 1:1000 dilutions, respectively. Blots were developed using a 1:2000 dilution of horseradish peroxidase-tagged goat anti-rabbit (Calbiochem, San Diego, CA) or anti-mouse (Vector Labs, Burlingame, CA) IgG antibody and the bands visualized using ECL™ (Amersham, Piscataway, NJ) reagent. Blots were scanned using a Canoscan Lide500F photo scanner and integrated optical densities of individual bands on scanned images were determined using Image J v.1.41 software (NIH).

### *In vitro *cell motility and invasion assays

Uncoated polyethylene terephthalate (PET) track etched membrane (24-well insert, pore size 8 μm; BD Biosciences, Franklin Lakes, NJ) inserts were equilibrated by adding 0.5 ml cell culture medium without FBS to the upper and lower chambers followed by incubation at 37°C for 2 h. The medium used for equilibration was aspirated gently and upper chambers were seeded with 10^5 ^cells in 0.5 ml of cell culture medium. TGF-β (100 pM) and/or 1D11 (10 μg/ml) or LY2109761 (2 μM) were added to both the upper and lower chambers. Following a 24-hour incubation at 37°C, cells in suspension were removed by washing twice with PBS and cells adherent to the top of the inserts removed by scraping the upper surface of the membrane with cotton tip applicators. The cells that had migrated to the underside of the inserts were fixed and stained using the Diff-Quick (Dade Behring, Newark, DE) staining kit as per manufacturer's instructions. Cells in ten random squares of 0.1 mm^2 ^in each well were counted at 200 × magnification, using 3 duplicate wells per assay condition, and expressed as number of cells per mm^2^. Invasion assays were carried out in an identical manner using Matrigel^® ^coated PET inserts (BD Biosciences, Franklin Lakes, NJ).

### Organotypic three-dimensional (3D) cultures

3D cultures were carried out as described by Debnath et al [60]. Briefly, 5000 cells were plated on top of solidified Growth Factor Reduced Matrigel^® ^(BD Biosciences, Franklin Lakes, NJ) in each well of an 8 well chamber slide. Cells were fed every other day with cell culture medium containing 2% (v/v) Matrigel^®^. Cells were washed with PBS on day 9 and fixed with buffered formalin for 10 minutes. For dose-response studies, cells were treated with vehicle (DMSO 0.28%), or with varying concentrations of LY2109761. All dilutions were made in cell culture medium supplemented with 10% (v/v) FBS and 2% (v/v) Matrigel^®^. Cells were fed every other day with vehicle and LY2109761. On day 9, cells were fixed and permeabilized using Triton-X 100 for 5 min, washed with PBS and incubated in the dark with Alexa Fluor 488 Phalloidin (1:40 dilution in PBS with 1% BSA, Invitrogen, Carlsbad, CA). The nuclei were stained using Topro-3 (1:150 dilution, Invitrogen) for 15 minutes. Stained slides were mounted with Prolong Antifade Reagent (Invitrogen, Carlsbad, CA) and photographed using a Zeiss epifluorescence microscope equipped with a MTI CCD camera and Nikon C1 confocal microscope. Volocity software (Improvision, Waltham, MA) or Huygens Professional software (Scientific Volume Imaging, Hilversum, Netherlands) renderer modules were used to generate perspective renderings of each image stack.

### Experimental metastasis assays

MDA-231-4175TR tumor cells were injected into the tail vein (2 × 10^5 ^cells) and MDA-231-SCP2TR (1 × 10^5 ^cells) and MDA-231-2860TR (5 × 10^5 ^cells) were injected into the left cardiac ventricle of viral antibody-free 4- to 5-week-old female athymic nude mice (Harlan Laboratory, Indianapolis, IN) to give rise to experimental lung and bone metastases, respectively [42,43]. Starting the following day, mice were treated with 5 mg/kg 1D11 anti-TGF-β antibody, 13C4 control antibody or buffer by intraperitoneal injection 3 times/week until tumor growth required sacrifice [61]. Alternatively, mice were treated with 50 mg/kg LY2109761 or 0.2 mL of vehicle by gavage twice a day, beginning on the second or third day following tumor cell inoculation, until the animals were sacrificed [62]. Body weight and bioluminescence were monitored weekly. For bioluminescence imaging (BLI), anesthetized mice were injected with 100 mg/kg d-Luciferin (Xenogen, Alameda, CA) in PBS intraperitoneally, and images were acquired using a Kodak 2000 MM Multimodal Imaging Station with cooled CCD camera (Carestream Molecular Imaging, New Haven, CT). Acquisition time was adjusted to avoid saturation of the signal. Analysis of the images was performed using Kodak Molecular Imaging Software Version 4.5 by first converting the signal to photon flux (measured in photons/sec/mm^2^), identifying regions of interest with a pixel density above background using the auto ROI feature of the software, and recording the sum of the background-subtracted pixel values within each ROI. Results are reported as bioluminescence per treatment group corrected for the number of mice per group. *Post mortem*, radiographic images from dissected forelimbs and hind limbs of the tumor bearing animals were taken using X-rays at 35 kVp for 8 seconds using a Faxitron LX-60 X-ray cabinet (Faxitron X-Ray, LLC, Lincolnshire, IL). The images were then used to quantify lesion areas using MetaMorph *7.5 *image analysis software (Molecular Devices, Sunnyvale, CA). Lung wet weight at the time of sacrifice was determined and expressed as a fraction of body weight. In addition, anterior and posterior photographic images of lungs were obtained from each animal *post mortem and the *fraction of lung surface occupied by metastases determined using NIH Image J (version 1.41) image analysis software. Besides lungs and bones, liver, kidneys, adrenal glands, and major lymph node groups were visually inspected for the presence of tumor metastases. Organs were fixed in formalin for 24 h and then placed in 70% ethanol until further histological assays were performed. In addition, uninvolved kidneys and lungs were snap frozen in liquid nitrogen for pharmacodynamic studies using RT-PCR and Western blot analysis.

### Cell proliferation-, apoptosis and angiogenesis

Tissue sections were deparaffinized, rehydrated, and stained with hematoxylin and eosin (H&E), rat antimouse monoclonal CD34 IgG2a (1:100; CL8927AP; Cedarlane, Hornby, Canada), or rabbit polyclonal anti-Ki67 (1:100; ab833-500; Novus Biologicals, Littleton, CO). Control slides were stained using appropriate isotope control antibodies. Biotinylated secondary antibodies (1:150; Zymed, San Francisco, CA) were used for detection. The total number of CD34-positive microvessels were counted in 5 randomly selected high-power (400 ×) fields in areas of viable tumor. To assess the percentage of proliferating cells, the proportion of Ki-67-positive nuclei was determined. At least 600 nuclei were counted in 5 randomly selected high-power (400 ×) fields in areas of viable tumor. Apoptotic cells were identified by terminal deoxynucleotidyl transferase-mediated nick-end labeling (TUNEL) assay using the *In Situ *Cell Death Detection Kit (Roche Molecular Biochemicals, Indianapolis, IN). To assess the degree of apoptosis, TUNEL-positive cells were counted in the tumor in 5 randomly selected high-power (400 ×) fields in areas of viable tumor.

### Histological staining for tartrate resistant acid phosphatase (TRAP)

For TRAP staining, bones were fixed in 10% (v/v) formalin followed by decalcification in 0.5 M EDTA. Slides were incubated with pre-warmed 10% (v/v) naphthol-ether (0.044 M 7-bromo-3-hydroxy-2-naphthoic-*o*-anisidide phosphate in ethylene glycol monoethyl ether) in basic incubation medium (0.112 M sodium acetate, 0.05 M disodium tartrate dihydrate) at 37°C for 30 minutes. Slides were then transferred directly into 2% (v/v) color reaction medium (1:1 mixture of 0.058 M NaNO_2 _and 0.154 M pararosaniline chloride in 2 M HCl in basic incubation medium), and incubated for 5 to 30 minutes at room temperature. Once optimal staining was achieved, slides were rinse in deionized water and counterstained using Harris's acid hematoxylin. The number of TRAP positive cells per mm of tumor adjacent to bone were used as a measure of osteoclast activity [27].

### Real-time quantitative RT-PCR

Transcript levels of individual genes were assayed in frozen tissue specimens by quantitative real time (qRT)-PCR, using the QuantiTect™ Probe RT-PCR Kit (QIAGEN, Valencia, CA). For the PCR, 50 μl reactions were set up with 100 ng of RNA, 0.4 μM primer, 0.2 μM dual labeled probe, 0.5 μl of QuantiTect™ Reverse Transcriptase Mix and QuantiTect™ Probe RT-PCR Master Mix. Real time PCR was performed using a Mx4000^® ^Multiplex Quantitative PCR System (Stratagene, La Jolla, CA) with each sample assayed in triplicate. Three mRNA species were quantified, including *CTGF *and *PAI-1 *and the reference gene, *GAPDH*. Standard curves for all three genes were generated using serial dilution of RNA isolated from tissue of control mice. The relative mRNA amounts for each of the genes in the individual RNA samples were calculated from the standard curves. The following primers and Taqman probes were used: *CTGF*: Forward Primer: 5'-aagggcctcttctgcgattt-3'; Reverse Primer: 5'-tttggaaggactcaccgctg-3'; Probe: 5'-/56-FAM/cctgtgtcttcggtgggtcggtgtac/3BHQ_1/-3'. *PAI-1*: Forward Primer: 5'-tgcatcgcctgccattg-3'; Reverse Primer: 5'-ggaccttgagataggacagtgctt-3'; Probe: 5'-/56-FAM/tggagggtgccatgggcca/3BHQ_1/-3'. *GAPDH*: Forward Primer: 5'-gtcgtggatctgacgtgcc-3'; Reverse Primer: 5'-gatgcctgcttcaccacctt-3'; Probe: 5'-/56-FAM/cctggagaaacctgccaagtatgatgacat/3BHQ_1/-3'

### Statistical analysis

One-way analysis of variance (ANOVA) tests and t-tests were performed using InStat (GraphPad Software, Inc., version 3.1a). Two-way repeated measures ANOVA tests and survival analyses were carried out using JMP (SAS Institute Inc., version 8).

## Competing interests

MR has received consulting and speaker fees from Genzyme Corporation and from Eli Lilly & Co. within the past five years.

## Authors' contributions

VG and RG carried out the bulk of the *in vitro *and *in vivo *studies. AG carried out the 3D culture studies. WX carried out the immunostaining studies. WB-P assisted with the *in vivo *studies, particularly bioluminescence assays, and carried out TRAP staining. YK provided all of the metastatic cell lines and invaluable advice in design and analysis of the *in vivo *experiments. SL and JM provided 1D11 and guidance for its use *in vitro *and *in vivo*. JMY provided LY2109761 and guidance for its use *in vitro *and *in vivo*. SB and GRM carried out the Faxitron and morphometric analyses carried out the immunoassays. MT participated in the sequence alignment. ES participated in the design of the study and performed the statistical analysis. MR conceived of the study, and participated in its design and coordination and helped to draft the manuscript. All authors read and approved the final manuscript.
